# Intelligent Real-Time Healthcare and Biomedical Monitoring Systems: A Narrative Review of AI, IoT, and Emerging Technologies

**DOI:** 10.3390/bioengineering13091065

**Published:** 2026-09-14

**Authors:** Abdussalam Elhanashi, Sergio Saponara

**Affiliations:** Department of Information Engineering, University of Pisa, 56122 Pisa, Italy; sergio.saponara@unipi.it

**Keywords:** artificial intelligence, internet of medical things, wearable sensors, deep learning, edge computing, TinyML, federated learning, blockchain, digital twin, explainable AI, remote patient monitoring, narrative review

## Abstract

Background: Chronic disease management, an ageing global population, and the aftermath of the COVID-19 pandemic have pushed real-time, continuous health monitoring from a research curiosity toward routine clinical practice. Artificial intelligence (AI), the Internet of Medical Things (IoMT), edge computing, and next-generation wireless networks are converging to enable systems that sense, interpret, and act on physiological data outside the traditional hospital setting. Methods: This is a narrative review, not a PRISMA-guided systematic or scoping review; it synthesises 75 sources (a mixture of primary studies, systematic/scoping reviews, and meta-analyses), individually verified against their published record. The topics span IoMT system architecture and security, wearable and implantable sensing, deep learning for electrocardiogram (ECG), fall-related and human-activity signal analysis, edge and TinyML deployment, federated learning, blockchain-based health-record security, medical imaging diagnostics, explainable AI (XAI), continuous glucose monitoring, 5G/6G-enabled telemonitoring, digital twins, consumer-grade and contactless cardiac sensing, neurological and mental health monitoring, and the materials, regulatory, and acute care infrastructure surrounding real-time deployment. Studies were organised into a five-layer architectural taxonomy spanning perception, edge, network, cloud, and application layers. Results: Reported accuracies for deep learning models on ECG arrhythmia classification range from 91% to 99.5% across the reviewed studies, but the figures come from different datasets, class definitions, and validation protocols and are therefore not directly comparable; within this heterogeneous evidence, edge-deployed models report accuracies in the 85–96% range at substantially reduced power budgets. Deep-learning-based fall detection and chest radiograph classification are each reported, in the individual studies reviewed, to outperform threshold-based or classical alternatives, though this has not been established through head-to-head comparison across the full evidence base. Federated learning and blockchain are discussed as technical mechanisms that can contribute to data privacy and record integrity; neither constitutes regulatory compliance with frameworks such as HIPAA or the GDPR on its own. Persistent obstacles identified across the reviewed literature include dataset heterogeneity, limited external clinical validation, energy-constrained edge hardware, low clinician trust in opaque models, and fragmented interoperability standards. Conclusions: The evidence reviewed here is consistent with, but does not by itself establish, a layered, privacy-preserving, and explainable architecture that couples lightweight on-device inference with federated or blockchain-secured cloud learning as a design direction for future real-time healthcare monitoring systems. Future work should prioritise standardised benchmarking, prospective clinical validation, and regulatory-aligned data–governance frameworks.

## 1. Introduction

Health systems worldwide are under sustained pressure. Population ageing, the rising burden of chronic disease, and workforce shortages all cut into hospitals’ capacity to keep patients under continuous observation. COVID-19 added momentum to a trend already underway, normalising remote consultation and pulling telemedicine infrastructure into the clinical mainstream.

Three technological threads have converged to make continuous, real-time monitoring of this kind practical outside hospital walls. One is the Internet of Medical Things (IoMT), which is a network of wearable, implantable, and ambient sensors that continuously pick up physiological signals and pass them along for analysis [1,2,3,4,5]. Another is artificial intelligence, specifically deep learning, which turns raw physiological time series and medical images into something clinically usable: an arrhythmia label, a fall alert, a glucose trajectory, a radiographic diagnosis; in individual studies, the accuracy is reported to approach manual interpretation for specific, narrowly defined tasks [6,7,8,9]. The third is less visible but no less essential: the computing, communication, and governance layer that makes the first two work at scale. Edge and TinyML inference keep the analyses close to the sensor [10,11,12,13]; federated learning and blockchain are technical mechanisms that let institutions train shared models without pooling sensitive records in one place [14,15,16,17,18]; 5G/6G-class connectivity handles low-latency transmission and localisation [19,20]; and explainable AI (XAI) methods try to make what the model outputs legible to the clinician reading it [21,22].

These threads tend to get reviewed one at a time. Surveys of wearable sensing lean toward materials and transduction mechanisms [23,24]; surveys of IoMT architecture lean toward network layers and threat models [1,2,3,4,5]; and surveys of federated learning or blockchain lean toward cryptographic guarantees [14,15,16,17,18]. What is harder to find is a single picture connecting the sensing layer, the inference layer, and the privacy layer, or a comparison that places reported accuracy figures for one clinical task alongside each other across both server-scale and edge-deployed versions, spanning modalities as different as ECG waveforms, chest radiographs, and continuous glucose traces. This review addresses that gap through a narrative synthesis rather than a systematic or scoping review; it does not follow a PRISMA-guided search-and-screening protocol, and Section 3 states this limitation explicitly rather than presenting the coverage below as exhaustive.

That is the gap this review tries to close. It proposes a five-layer architectural taxonomy, running from physical sensing through to clinical application, that organises the technologies discussed in the literature into one coherent system view and that Section 2.1 explicitly positions against existing IoMT reference architectures rather than presenting as free-standing. It pulls together quantitative findings from the reviewed studies on wearable and IoMT-based monitoring, ECG-based deep learning, edge/TinyML deployment, privacy-preserving collaborative learning, medical imaging diagnostics, explainable AI, continuous glucose monitoring, and connectivity/digital twins. Additionally, it distils the technical and translational barriers that keep surfacing across these studies into a discussion of open challenges, review limitations, and future directions.

The main contributions of this review can be summarised as follows:

- **Architectural taxonomy:** It proposes a five-layer taxonomy—perception, edge/fog, network, cloud/platform, and application—that organises 75 individually verified sources, comprising primary studies and secondary reviews (Table 1), spanning IoMT, wearable sensing, deep learning, edge/TinyML, federated learning, blockchain, medical imaging, explainable AI, and connectivity into a single coherent system view that is explicitly compared against existing IoMT reference architectures rather than presented as free-standing.
- **Cross-study quantitative synthesis:** It consolidates quantitative evidence on cardiac biosignal classification, presenting server-scale and edge-deployed model accuracy alongside dataset, sample size, and computational cost information where reported, while explicitly flagging that these figures are not directly comparable across studies.
- **Extended application coverage:** It extends the analysis beyond the wearable and cardiac core that dominates prior reviews into ten further application areas, medical imaging, explainable AI, continuous glucose monitoring, consumer-grade and contactless sensing, neurological and mental health monitoring, and materials/systems/acute care infrastructure, with the conceptual boundaries of each extension stated explicitly in Section 3.2.
- **Structured challenges, limitations, and future directions:** It distils the technical, regulatory, and translational barriers recurring across this literature, such as the dataset heterogeneity, scarce prospective validation, energy-constrained hardware, and limited regulatory transparency, into a structured discussion that separates evidence-supported observations from proposed future directions and states the methodological limitations of this narrative review alongside those of the underlying evidence base.

Section 2 surveys related work by technological layer and application domain; Section 3 sets out the review methodology, including source classification, scope boundaries, and the reasons a formal risk-of-bias appraisal was not performed; Section 4 presents the synthesised results, opening with the characteristics of the included evidence base before moving to thematic synthesis; Section 5 discusses cross-cutting challenges and the limitations of the current evidence base; and Section 6 concludes the paper.

**Table 1:** Classification of the 75 sources included in this review by publication and evidence type.

Category	Count	Description
Primary empirical study	19	Original research reporting a specific model, device, or clinical outcome
Systematic review/scoping review/meta-analysis	32	Secondary evidence with a stated, reproducible search and selection methodology
Narrative/general review	23	Secondary evidence without a systematic search protocol
Methodology guideline	1	PRISMA 2020 statement, cited for methodological terminology

## 2. Related Work

### 2.1. Internet of Medical Things Architectures and Security

The IoMT literature converges on a broadly similar layered description of the technology stack, even though terminology differs from one author to the next. Sindhuja et al. describe the IoMT as the interconnection of medical devices, sensors, applications, and systems that enables real-time collection, transmission, and analysis of patient data and the cataloguing of applications spanning smart hospitals, telemedicine, fitness tracking, and remote patient monitoring [1]. He et al. add an explicit treatment of lightweight and explainable AI methods suited to constrained IoMT nodes, along with a structured account of data security and privacy mechanisms across the IoMT data lifecycle [2]. Khan et al. zero in on the authentication layer specifically, evaluating IoMT authentication protocols against requirements such as mutual authentication, forward secrecy, and computational cost using formal verification tools [3]. Koutras et al. classify IoT communication protocols by where they sit—perception, network, or application layer—and find that several widely used IoMT protocols, RFID and certain Bluetooth profiles among them, lack adequate built-in encryption and authentication, leaving them open to eavesdropping and man-in-the-middle attacks [4]. Al Khatib et al. take the broadest view of applications, cataloguing mobile health apps, RFID-based hospital logistics, IoT-based disease prediction, and searchable-encryption or blockchain-based health-record sharing as concrete deployed use cases while flagging privacy protection, sustainable power sources, and device reliability as the most persistent open problems [5].

Taken together, these five surveys point toward the same perception–edge–network–cloud–application taxonomy adopted in Section 3 here: each addresses a different horizontal slice of the same stack, and each lands independently on security and energy constraints as the dominant unresolved issues. This five-layer split is not presented here as an original invention; it is a refinement of existing IoMT and IoT reference architectures. Widely used three-layer IoT models (perception, network, application) divide the stack more coarsely, and several of the IoMT surveys cited above already separate perception, network, and application layers explicitly [1,2,3]. The refinement made here is to split the network layer into an edge/fog sub-layer (local, on-device inference) and a network sub-layer proper (data transport) and to add an explicit application layer for clinician- and patient-facing interpretation; this split is useful for organising the specific technologies covered in this review, that is, TinyML inference versus 5G/6G transport versus explainable-AI interfaces, but it is an organisational device for this synthesis rather than a validated architectural standard.

The security posture of this stack deserves a more granular treatment than a single statement that security is a challenge. Different threat types map onto different layers: data interception and eavesdropping primarily target the network layer, where several IoMT protocols lack built-in encryption [4]; device compromise and physical tampering target the perception layer, where wearable and implantable sensors typically have minimal on-device security hardware; model poisoning and data poisoning target the cloud/platform layer during federated training [14]; membership-inference and model-inversion attacks target the same layer during or after training, attempting to reconstruct training data from model parameters or updates; and adversarial perturbation attacks target the edge/application boundary, where a crafted input to a deployed inference model can trigger an incorrect classification. Table 11 in Section 4.9 maps these threat categories onto the five-layer architecture explicitly.

### 2.2. Wearable and Implantable Sensing

Wearable sensing constitutes the physical basis of the monitoring systems discussed throughout this review. Shajari et al. survey physical, chemical, and biosensor categories of wearable devices and argue that flexible electronics become clinically useful only once paired with AI-based signal interpretation, since it is the AI component that compensates for the noise and motion artefacts inherent to skin-mounted sensing [23]. Cheng et al. address the materials side of the same problem, covering substrate and functional materials, piezoresistive and triboelectric transduction mechanisms, and the system integration choices that keep sensors reliable during ordinary daily activity [24]. Abedi et al. conducted a scoping review of AI-driven, real-time cardiovascular monitoring with wearable devices and found that only 19 of 153 full-text studies assessed met their inclusion criteria for genuinely real-time, clinically validated deployment, concluding that the field remains under-validated despite the promise of the underlying technology [25]. Gu et al.’s PRISMA-guided systematic review of 30 studies on wearable sensors for clinical applications, rehabilitation, and disease risk assessment reported areas under the ROC curve as high as 0.97 for fall risk and mobility decline prediction while identifying small sample sizes and inconsistent methodology as recurring limitations [26]. Aruleba’s narrative review proposes a translational framework consisting of a validation ladder progressing from offline feasibility to real-world workflow performance, together with a reliability toolkit for calibration, uncertainty-aware thresholds, and drift monitoring, intended to make edge-deployed wearable models safer to update after deployment [27].

### 2.3. Fall Detection and Human Activity Recognition

Fall detection and human activity recognition (HAR) are probably the most heavily studied applications of wearable AI monitoring, and the trade-offs they surface recur throughout the rest of this review. Gorce and Jacquier-Bret’s PRISMA-guided systematic review spans 80 studies across seven databases, comparing wearable, non-wearable (ambient/vision), and hybrid sensor categories against threshold-based, classical machine learning and deep learning detection methods; deep learning came out ahead on accuracy, precision, sensitivity, specificity, and F1-score in the studies they screened, while wearable-only configurations underperformed hybrid and non-wearable setups on the same metrics [28].

Nait Aicha et al. used trunk-worn accelerometer recordings from 296 older adults to compare convolutional, recurrent, and hybrid convolutional–recurrent architectures against a hand-engineered biomechanical baseline for fall risk prediction and found that automatically learned features could match or exceed features designed by domain experts [29]. Mohammad et al. proposed a wearable pre-impact fall detection framework consisting of a CNN-RNN ensemble intended to trigger a protective airbag before ground impact rather than issue an alarm after the fact, illustrating a shift in this sub-field toward intervention rather than passive detection [30]. Nait Aicha et al. and Mohammad et al. are cited here as illustrative primary studies that may already be indirectly represented within the 80-study evidence base synthesised by Gorce and Jacquier-Bret. This review does not attempt to deduplicate participant-level data across primary studies and the systematic reviews that may already include them, since the citations here serve a technology-mapping purpose rather than a pooled-effect-estimate purpose (see Section 3.3).

At the broader level of activity recognition research underlying fall detection, Gu et al.’s widely cited ACM Computing Surveys article catalogues deep learning approaches to HAR across sensor modalities and model families and identifies two problems that recur throughout this review: the expense of collecting large labelled datasets, and the difficulty of running deep HAR models on the resource-constrained smartphones and smartwatches where the sensor data originate [31].

### 2.4. Deep Learning for Cardiac Biosignal Monitoring: ECG and Beyond

Electrocardiogram (ECG) interpretation is the most mature application of deep learning in real-time cardiac monitoring, and the literature reviewed here spans everything from maximal server-side accuracy down to a minimal edge-device footprint. This subsection covers eight studies in total: seven address ECG signals specifically [6,7,8,10,32,33,34], and one addresses a related but distinct cardiac biosignal, namely, the phonocardiogram (heart sound) [11]; these two signal types are discussed together because they share the same edge-deployment trade-offs, but they are not the same modality, and Table 5 marks the distinction explicitly.

At the accuracy-maximising end, Khan et al. built a one-dimensional convolutional residual network, trained on the PhysioNet MIT-BIH Arrhythmia Database with synthetic minority oversampling to handle class imbalance, and reported 98.63% average accuracy across five heartbeat classes under ten-fold cross-validation [7]. Zheng et al. took a different route, combining multi-resolution short-time Fourier transform inputs with a squeeze-and-excitation attention mechanism in a dual-branch convolutional architecture; their reasoning was that fusing short- and long-segment time-frequency representations captures morphological and rhythmic information that a single-resolution model would miss, and they reported an overall accuracy of 96.0% on their evaluation set [6]. Jiang et al.’s HADLN architecture pairs a residual network with a bidirectional LSTM and an attention mechanism, tested on the PhysioNet 2017 Challenge dataset for four-class rhythm classification; they reported an overall accuracy of 91.0% and noted that the attention mechanism also makes the model’s decisions easier to interpret [32]. Bai et al., meanwhile, built a hybrid convolutional and bidirectional gated recurrent unit network with multi-head attention for 12-lead ECG classification, testing it across both MIT-BIH and the PTB Diagnostic ECG database and reporting multi-class performance in terms of AUC and F1-score rather than a single overall accuracy figure, since their task involved multiple simultaneous diagnostic labels per recording [8].

At the resource-constrained end sit three studies that test just how much accuracy survives aggressive compression. Elsheikhy et al.’s lightweight convolutional architecture, built around a channel attention mechanism and able to run on both 2-lead and 12-lead ECG signals, reached 99.18% accuracy on MIT-BIH and 99.48% on INCART [33]—figures that hold up well given the reduced footprint but that were obtained on different databases from the studies above and are not a like-for-like comparison. Wang et al. extended this approach by binarising the weights and activations of a compact convolutional network; they reported 95.67% accuracy against a 96.45% full-precision baseline, a reduction of 0.78 percentage points, in exchange for a 12.65 times computational speedup and a 24.8 times reduction in storage, a trade explicitly aimed at wearable hardware where memory and power are the binding constraints [34]. Hizem et al. deployed a pruned and quantised TinyML model directly on Raspberry Pi and Arduino-class devices for ECG anomaly detection; the result was 92.3% accuracy at 0.024 mW [10]. A structurally similar result outside ECG proper comes from Ma et al., whose lightweight convolutional network for phonocardiogram-based valvular heart disease screening uses ten times fewer parameters than comparable deep learning models; combined with self-supervised pre-training, it reaches 4–20% higher classification accuracy on noisy or perturbed data than an equivalent non-self-supervised model and completes on-device inference on a standard smartphone in 0.03–0.37 s, several times faster and using roughly a third of the power of a full-size CNN with ten times the parameters [11].

Read across these eight studies, the accuracies cluster in the high-90s for studies without an explicit hardware constraint and in the 85–96% range for studies explicitly targeting edge or resource-constrained deployment. This pattern should be read as an observation drawn from a heterogeneous set of studies using different datasets, class counts, and validation protocols, not as a controlled comparison demonstrating that server-scale models are inherently superior to edge-deployed ones, or as a measured accuracy cost of edge deployment; Section 4.4 and Table 5 return to this point and its limits explicitly.

### 2.5. Edge Computing and TinyML

TinyML, the practice of running inference on microcontroller-class hardware, enables the on-device results discussed in Section 2.4. Elhanashi et al. survey the tools, model-compression techniques (pruning, quantisation, knowledge distillation), and application domains of TinyML and identify healthcare monitoring as an application area where local inference offers two distinct benefits, namely, reduced data transmission requirements and lower end-to-end latency, since continuous physiological data do not need to leave the device that captured it [12].

Heydari and Mahmoud took a broader, PRISMA-guided look at TinyML experimental studies, restricting their scope to work that actually demonstrated inference on an embedded device rather than merely proposing a compression method. They found typical inference latencies of 0.18–300 ms, comfortably under the 10–500 ms range usually required for something to count as real-time, though they also caution that most of the studies they surveyed under-report how model size, task complexity, and the target device’s memory ceiling interact [13]. This is broadly consistent with the accuracy-versus-footprint pattern described for the ECG and heart murmur studies in Section 2.4, though the two studies use different outcome metrics (latency versus accuracy) and were not compared directly by either set of authors.

### 2.6. Federated Learning and Blockchain for Privacy-Preserving Collaboration

Centralising physiological data from many patients and institutions for model training conflicts with regulatory frameworks such as HIPAA and the GDPR and concentrates breach risk. Federated learning (FL) is a technical mechanism that addresses part of this problem by training a shared model across decentralised data holders without moving the underlying data; it is not by itself a certification of regulatory compliance, since HIPAA and GDPR compliance depend on organisational, contractual, and governance measures well beyond the training algorithm. Abbas et al. review FL’s integration with IoT devices, wearables, and remote-monitoring systems in smart healthcare and catalogue the security threats specific to this setting, including adversarial attacks, data poisoning, and model-inversion attacks that attempt to reconstruct training data from the model updates themselves [14]. Mir et al. take a systematic review approach to the ethical side of FL in healthcare; screening six databases under PRISMA guidelines and keeping 38 of 3047 initially identified records, they find medical imaging and electronic health records to be the domains in which FL is most frequently applied, while algorithmic fairness and governance remain comparatively unexamined relative to privacy mechanisms [15].

Blockchain tackles a related but separate problem: keeping health records intact, auditable, and under patient control once they exist, regardless of whether the model training itself is federated. Qammar et al. systematically reviewed the combination of the two, arguing that blockchain’s immutable ledger can secure the aggregation step of federated training and provide an audit trail that FL alone cannot offer [16]. Ettaloui et al. worked through 31 blockchain-based electronic health record (EHR) architectures pulled from over 600 candidates, scoring them on privacy, security, authentication, availability, data control, storage, and resource consumption; decentralised storage and fine-grained access control came out as the most consistent benefits, with transaction throughput and storage overhead as the most consistent costs [17]. Jayapriya and Jeyanthi reach a similar view of state-of-the-art blockchain schemes for EHR management, framing blockchain as an answer to a familiar problem: patient records scattered across institutions, forcing redundant testing every time someone changes provider [18]. As with federated learning, these mechanisms should be read as technical contributions to data governance rather than as regulatory compliance themselves; none of the cited studies claims otherwise, but the distinction is easy to lose when summarising across studies and is therefore stated explicitly here.

### 2.7. Deep Learning for Medical Imaging Diagnostics

Beyond continuous physiological signal monitoring, deep learning has also found heavy use in episodic diagnostic imaging, especially chest radiography and computed tomography. This falls outside a strict definition of continuous or real-time physiological monitoring and is included here because point-of-care chest imaging is frequently acquired and interpreted within an acute, time-critical clinical workflow (for example, ruling out pneumonia or COVID-19 in an emergency department triage setting); Section 3.2 states this scope decision, and the corresponding conceptual boundary, explicitly rather than leaving it implicit.

Al-Sheikh et al. built a lung disease classification pipeline around a novel image enhancement pre-processing step feeding into a customised convolutional network plus two pre-trained architectures, AlexNet and VGG16, tested on chest X-ray and CT scans for telling COVID-19, pneumonia, and normal lungs apart [9]. Meedeniya et al.’s systematic review of deep learning for chest X-ray analysis, focused on pneumonia and COVID-19 detection, found that architectures pairing a deep convolutional feature extractor with a separate, sturdier machine learning classifier tended to beat purely end-to-end designs with a fully connected classification head [35]. Both studies point at the same pattern already visible in the ECG literature: pre-processing quality and hybrid architectural choices seem to matter just as much as raw model depth, though neither strand of literature has been benchmarked against the other directly.

### 2.8. Explainable AI for Clinical Decision Support

A caveat runs through the deep learning literature covered in Section 2.4 and Section 2.7: high accuracy by itself does not guarantee clinical adoption if the model’s reasoning stays opaque to the clinician who has to act on it. Abbas et al.’s systematic meta-analysis of 62 peer-reviewed studies on explainable AI (XAI) in clinical decision support, spanning 2018 to 2025, finds that Grad-CAM is dominant in imaging tasks and that attention mechanisms are dominant in sequential data tasks, alongside persistent gaps in longitudinal clinical validation and standardised interpretability metrics [21]. Xu et al.’s earlier systematic review of CDSS interpretability, covering 2011 to 2020, draws a line between ante hoc approaches, i.e., models that are transparent by construction, decision trees, and fuzzy logic, and post hoc approaches, with explanations added on after training via feature-importance or saliency methods, and argues that the right choice depends on both the data type and what the clinician and patient actually need [22]. Set against the attention-based ECG models covered in Section 2.4 [6,32,33], this suggests, as a hypothesis rather than an established finding, that architectural interpretability, specifically attention weights visualised directly over an ECG trace, may earn clinical trust more readily than post hoc explanation of an otherwise black-box model; none of the cited studies tested this comparison directly.

### 2.9. Continuous Glucose Monitoring and Metabolic Digital Phenotyping

Continuous glucose monitoring (CGM) is one of the oldest forms of continuous physiological monitoring in clinical use, which makes it a useful benchmark for the newer sensing modalities covered elsewhere in this review. Kapoor and Hasija’s meta-analysis of machine learning models trained on CGM and other IoT device data for blood glucose prediction, which included ten eligible studies across 15- to 60-min prediction horizons, found that prediction error climbs steadily with horizon length: the mean RMSE rose from 15.02 mg/dL at 15 min to 35.89 mg/dL at 60 min, with Random Forest being the strongest performer among the algorithms compared [36]. Metwally et al. approached the same sensor stream from a different angle altogether. Rather than predicting long-term trends, they used CGM-derived glucose curves from at-home oral glucose-tolerance tests to classify prediabetic individuals into distinct metabolic subphenotypes—muscle insulin resistance, hepatic insulin resistance, beta-cell dysfunction, and impaired incretin action—reporting areas under the curve of 88–95% and arguing that this form of digital phenotyping could support more individualised prevention than the current binary prediabetes/diabetes cutoff allows [37]. Between them, these two studies indicate a second axis of value beyond the accuracy-versus-power trade-off that dominates the ECG literature: the same continuous sensor stream can support both short-horizon predictive alerting and longer-horizon phenotypic classification, depending on how the data are modelled.

### 2.10. Fifth- and Sixth-Generation Networks and Digital Twins

Two more technologies extend the monitoring architecture beyond the sensor–edge–cloud pipeline. Batool built a deep learning architecture consisting of convolutional and LSTM layers plus an attention mechanism, explicitly co-designed with 5G ultra-reliable low-latency communication (URLLC) for real-time vital sign prediction, and reported stable performance across a three-month evaluation with 1000 patients and thousands of concurrent monitoring sessions [19]. Looking a generation further out, Trevlakis et al.’s comprehensive survey of localisation in 6G wireless systems covers millimetre-wave, terahertz, and visible-light positioning models; it is important to note that 6G networks are not yet commercially deployed, and the localisation capabilities described are evaluated through simulation and modelling rather than field deployment [20] in existing hospital IoMT settings.

Khoshfekr Rudsari et al. review digital twin technology in healthcare, defining a digital twin as a continuously updated, patient-specific computational model drawing on imaging, genomic, and wearable sensor data; among the clinical outcomes they report from the underlying primary literature is a documented reduction of over 13% in cardiac arrhythmia recurrence following digital twin-guided ablation planning in one specific study, alongside ongoing challenges in computational scalability and validation across the wider field [38]. Banoub et al. carry the digital twin concept into ophthalmology specifically, reviewing case studies where DTs integrating genetic, environmental, and real-time patient data improved risk assessment and surgical planning for glaucoma and age-related macular degeneration while flagging data integration and privacy concerns as the main obstacles to clinical translation [39]. Both digital twin reviews describe a technology that remains predominantly at the modelling and early clinical evaluation stage rather than one in routine use; this qualification is stated here explicitly because digital twins are frequently discussed in aspirational terms in the broader literature.

### 2.11. Consumer-Grade Cardiac Sensing and Contactless Vital Sign Capture

A parallel strand of the literature looks at monitoring modalities that sit outside the clinical-grade wearable devices discussed in Section 2.4, either because they are already in mass consumer use or because they remove skin contact altogether. Shahid et al.’s systematic review and diagnostic meta-analysis of the Apple Watch electrocardiogram for atrial fibrillation, pooling 11 studies and 4241 participants, found a pooled sensitivity of 94.8% and specificity of 95% against standard 12-lead ECG, with an area under the ROC curve of 0.96. This pooled meta-analytic result is a strong signal for the atrial fibrillation detection use case specifically, but it does not establish consumer smartwatch ECG as diagnostic-grade in a general sense: the meta-analysis pools heterogeneous studies with varying reference standards, and the finding is specific to atrial fibrillation rather than to arrhythmia detection broadly [40].

A related but distinct problem, blood pressure, still resists a consumer-grade cuffless solution: Liu et al.’s two-branch ResNet-BiLSTM architecture for PPG-based cuffless blood pressure estimation, tested on the MIMIC-IV dataset, achieved mean absolute errors of 3.47 mmHg (systolic) and 2.81 mmHg (diastolic), meeting AAMI standards [41] and reaching a British Hypertension Society grade A rating [42], a result that suggests cuffless BP estimation can approach clinical-grade accuracy under the tested conditions, though this is a single-dataset result rather than a multi-cohort validation. A third sensing modality removes the wearable altogether: Kebe et al.’s review of radar-based vital sign detection covers both continuous-wave and ultra-wideband approaches to contactless heart rate and respiration monitoring and concludes that radar-based systems can match the accuracy of contact sensors for children and elderly patients specifically, which are populations where electrode attachment is often impractical or poorly tolerated [43].

### 2.12. Neurological, Mental Health, and Movement Disorder Monitoring

Continuous monitoring extends well beyond cardiac and metabolic signals into neurological and psychiatric applications, each of which reproduces the accuracy-versus-validation tension already visible elsewhere in this review. Abd-Alrazaq et al.’s systematic review and meta-analysis of wearable AI for sleep apnoea detection, drawing on 38 included studies, found that the pooled accuracy varied considerably by sensor placement and by which specific apnoea metric was being predicted, underscoring how sensitive this application is to device design choices rather than to the underlying AI model alone [44]. Singh et al.’s narrative review of deep learning applied to EEG, MRI, and wearable devices for epileptic seizure detection highlights that recent advances in DL have begun to enable real-time seizure detection directly on neural implants and wearables, a shift from the retrospective, offline classification that dominated earlier seizure-detection research [45]. In movement disorders, Pardoel et al.’s widely cited review of wearable-sensor-based detection and prediction of freezing of gait in Parkinson’s disease, covering 74 included studies, found that detection performance has improved steadily over time but that prediction, that is, anticipating a freeze before it happens rather than merely detecting one in progress, remains the harder and less mature problem [46]. A structurally similar gap between detection and earlier, more actionable prediction appears in Alhakeem et al.’s systematic review of AI-powered wearable technologies for stroke prediction, which identified only five studies meeting the inclusion criteria for real-time stroke risk prediction specifically, a small evidence base that the authors attribute to the practical difficulty of capturing a genuine pre-event physiological signature outside a hospital setting [47]. Turning to psychiatric applications, Abd-alrazaq et al.’s scoping review of wearable AI for anxiety and depression, screening 1203 candidate studies down to 69 included ones, found that most studies used wearable AI for diagnosis rather than treatment and called for more rigorous evaluation of diagnostic performance before wearable AI can be considered a reliable clinical tool in this domain [48].

### 2.13. Materials, Infrastructure, and Cross-Cutting Applications

A final cluster of studies addresses the materials science, healthcare system infrastructure, and specialised clinical applications that support or extend the core monitoring pipeline discussed throughout this review. On the sensing hardware side, Shao et al.’s review of carbon-based textile sensors for physiological signal monitoring, screening over 7500 candidate articles down to 85 representative studies, found a steadily rising publication rate for flexible textile sensors, evidence that garment-integrated sensing is maturing alongside the skin-patch and wristband forms more commonly discussed elsewhere in this review [49]. Rayegani et al.’s review of piezoelectric and triboelectric nanogenerators for self-powered wearable sensors addresses a problem that cuts across every wearable application discussed here: battery life. Their review catalogues hybrid nanogenerator designs intended to harvest ambient mechanical energy from the body itself, potentially reducing or eliminating the charging burden that limits continuous long-term monitoring [50]. Martins et al.’s review of non-invasive optical and microwave glucose biosensors covers near-infrared, Raman, and microwave dielectric sensing as alternatives to the CGM approaches discussed in Section 2.9 while noting that tissue heterogeneity and signal interference have so far kept these fully non-invasive methods short of the accuracy achieved by subcutaneous CGM sensors [51].

On the systems side, Zieni et al.’s interdisciplinary review of ambient assisted living (AAL) systems for home health monitoring frames privacy, cost, usability, and reliability as a persistent four-way trade-off in elderly home-monitoring deployments, a framing that echoes the layered privacy-versus-latency-versus-interoperability trade-off developed independently for the IoMT architecture in Section 4.1 [52]. Tan et al.’s systematic review of remote patient monitoring (RPM) interventions during the transition from inpatient to home care, covering 29 studies from 16 countries, found a consistent downward trend in hospital readmission, length of stay, and non-hospitalisation costs, providing outcome-level evidence for the clinical value of the monitoring architectures discussed throughout this review [53]. Cloß et al.’s scoping review of wearable applications in oncology patients, covering 112 studies, found that only 9.8% were randomised controlled trials and that most wearable use in cancer care remains at an early feasibility stage, a validation gap consistent with Abedi et al.’s findings for cardiovascular wearables in Section 2.2 [54]. Sarpong et al.’s review of smart wound dressings integrating pH, temperature, moisture, and bacterial sensors reports that these devices can detect infection-related changes earlier than routine visual inspection, though clinical validation again lags behind the underlying sensing technology [55]. Motahari-Nezhad et al.’s scoping review of systematic reviews of digital biomarkers found that most existing clinical trial evidence concentrates on physical activity and cardiac rhythm biomarkers specifically, leaving the broader space of digital biomarkers relatively under-studied at the level of randomised evidence [56].

Two further studies address infrastructure and workflow rather than sensing itself. Almarie et al.’s cross-sectional analysis of ML-enabled medical devices authorised by the US FDA in 2024 found that only 29.2% of device summaries reported both sensitivity and specificity, and only 16.7% included a Predetermined Change Control Plan for managing post-market model updates, which is a regulatory transparency gap that sits alongside the clinical validation gaps identified throughout this review [57]. Li et al.’s narrative review of cyber-attacks on hospital systems found that reported incidents have roughly doubled since the COVID-19 pandemic, reinforcing the security concerns raised for IoMT architectures in Section 2.1 and for blockchain-based record protection in Section 2.6 [58]. Robotic surgery is included here as a further example of real-time, sensor-driven monitoring applied intraoperatively, extending the scope of this review beyond ambulatory and home settings; Knudsen et al.’s review of the clinical applications of AI in robotic surgery documents how AI-assisted surgical systems increasingly incorporate continuous sensor feedback loops structurally similar to the wearable-monitoring pipelines discussed throughout this review [59]. Gao et al.’s review of deep-learning-based medical image segmentation methods, covering CNN, transformer, and foundation model approaches such as the Segment Anything Model, extends the imaging diagnostics discussion in Section 2.7 into the specific sub-task of delineating regions of interest within an image rather than classifying the image as a whole [60]. Large language models are included for a related reason: Artsi et al.’s systematic review of LLMs in real-world clinical workflows found that, despite an explosion of LLM research in medicine in general, only four studies met their criteria for genuine real-world clinical implementation as of April 2025, which places LLM-based clinical documentation and decision support at broadly the same early translational stage as several of the real-time monitoring technologies discussed elsewhere in this review [61]. Each of these three topics, namely, robotic surgery, image segmentation, and LLM workflows, is included as an adjacent or enabling technology rather than as a core real-time physiological monitoring application and is treated at correspondingly less depth than the wearable- and cardiac-monitoring core of this review.

A last set of four studies rounds out the acute care and specialised-sensing picture. Moor et al.’s systematic review of machine learning for early sepsis prediction in the ICU found that models combining multiple physiological streams consistently outperformed single-marker approaches in the studies they screened, though comparability across studies was limited by inconsistent sepsis-onset definitions [62]. Nguyen et al.’s before-and-after study of virtual ICU implementation across multiple critical care units found measurable improvements in clinical outcomes following deployment of a nurse-led, continuously monitored tele-critical-care programme [63]. Senechal et al.’s systematic review of non-contact and wireless vital sign monitoring in the neonatal intensive care unit, covering 60 observational studies, found that most emerging technologies still monitor only a single vital sign with offline data processing, leaving genuinely real-time, multi-parameter neonatal monitoring an open problem [64]. Finally, Sang et al.’s accelerometer-based wearable patch for respiratory rate and wheeze detection, aimed at asthma and COPD, reached 95% accuracy, 96% sensitivity, and 93% specificity with an AUC of 0.99 in their reported evaluation, outperforming a deterministic time-frequency baseline on the same dataset [65].

### 2.14. Data Quality, Equity, and Real-World Adherence

A final thread running across this literature, rather than confined to any one sensing modality, concerns whether a technically accurate system actually works, and works fairly, once it leaves the validation dataset. Ueda et al.’s review of fairness in healthcare AI proposes a set of Fairness of Artificial Intelligence Recommendations (FAIR) and traces algorithmic bias in imaging and diagnostic AI to data bias, design bias, and biased human–AI interaction, a taxonomy directly relevant to the imaging and ECG models discussed in Section 2.4 and Section 2.7, most of which are trained on demographically under-described public datasets [66]. Thapa et al.’s survey of security risks and user perceptions toward wearable IoMT adoption complements the technical security literature in Section 2.1 with the user side of the same problem, finding that privacy concern is among the strongest predictors of reluctance to adopt wearable medical devices in the first place [67]. Tun et al.’s systematic review of healthcare worker trust in AI-based clinical decision support systems identifies algorithmic opacity, insufficient training, and workflow disruption as the dominant barriers to adoption and explainability, usability, and clinical reliability as the dominant enablers, directly reinforcing the case made for architecturally embedded interpretability in Section 2.8 [68].

Two further studies address the practical reliability of the continuous data streams on which this review’s models depend. Frodi et al.’s analysis of long-term wearable adherence in the SafeHeart implantable cardioverter defibrillator cohort found a median adherence of 88.2% over six months, with older age and fewer device changeovers associated with better adherence; this provides concrete evidence that the model accuracy reported on curated datasets may not reflect the adherence-degraded data a deployed system actually receives [69]. Chakrabarti et al. address this data-degradation problem directly, comparing imputation methods for missing time-series data from wearable trackers and finding that imputation restricted to a time-local bin around the gap consistently outperformed whole-dataset imputation, a practical mitigation for a missing data problem that adherence studies such as Frodi et al.’s indicate is common rather than exceptional [70]. Two additional studies extend this focus on equity and reliability to specific populations and hardware directions. Hyun et al.’s scoping review of wearable biosensors for paediatric hospitals, covering 79 studies, found that only two-thirds of devices used in neonatal and paediatric care reported validated accuracy or reliability, indicating a weaker evidence base than the adult-focused studies that dominate this review [71]. Furthermore, Maita et al.’s scoping review of digital health solutions in rural areas found that connectivity and digital literacy barriers, rather than the underlying sensing or AI technology, were the most consistently reported obstacle to extending remote monitoring to underserved populations, factors that compound the demographic representation gaps identified by Ueda et al. above [72]. Lage Cañellas et al.’s self-supervised multimodal fusion framework, which combines heart and respiration waveforms from mmWave radar, RGB camera, and depth-camera sensors using contrastive learning, illustrates a related direction directly: with only 1% of the data labelled, their fused model reached 64% accuracy on breathing pattern classification against 24% for an equivalent fully supervised model trained on the same limited labels, indicating that self-supervised, multi-sensor fusion can substantially reduce the labelled data burden for wearable and contactless monitoring alike [73]. Giorgetti and Pau’s review of the transition from TinyML to edge generative AI points toward a related near-term direction for the edge-AI architecture discussed in Section 2.5: running larger generative and multimodal models directly on the resource-constrained devices already responsible for the ECG and TinyML results, as summarised in Table 5 [74].

## 3. Materials and Methods

### 3.1. Review Type and Search Approach

This is a narrative review, not a PRISMA-guided systematic or scoping review [75], and it does not claim to have the exhaustive, reproducible coverage that term implies. It followed an iterative, topic-driven search across peer-reviewed journal articles, systematic reviews, and conference proceedings indexed in PubMed/PMC, MDPI journals (Sensors, Electronics, Healthcare, Bioengineering, and Informatics, among others), Nature Scientific Reports and Nature Biomedical Engineering, PLOS ONE, Frontiers, JMIR, Elsevier, and IEEE venues, using a general purpose web search tool rather than direct queries against bibliographic databases such as PubMed, Scopus, or Web of Science. Search terms combined “real-time health monitoring”, “wearable sensors”, “Internet of Medical Things”, “deep learning”, “edge computing”, “TinyML”, “federated learning”, “blockchain”, “5G”, “6G”, “digital twin”, and “explainable AI” with clinical qualifiers such as “ECG”, “arrhythmia”, “fall detection”, “chest X-ray”, and “glucose monitoring” and were run between July and September 2026.

Consistent with this narrative approach, and in direct response to reviewer feedback on the initial submission, this review does not provide a PRISMA-style flow diagram with counts of records identified, screened, and excluded, because no database-level export or reproducible search log of that kind was generated: searches were run iteratively and refined based on what earlier searches returned, rather than executed once against a fixed set of databases with a pre-registered query string. Presenting a flow diagram with fabricated or reconstructed counts would misrepresent the process that was actually followed, so this limitation is stated directly here and revisited in Section 5.4 rather than concealed behind a diagram implying a reproducible, database-level search that did not take place.

### 3.2. Scope and Conceptual Boundaries

“Real-time” and “continuous” healthcare monitoring are used here to mean systems that acquire physiological or clinical data on an ongoing or repeated basis and process it with latency periods short enough to support a timely clinical or self-management response, typically seconds to minutes for physiological signals such as ECG or falls, and hours for slower-changing signals such as glucose trends. Under this definition, the core of this review is wearable, implantable, and ambient sensing paired with on-device or near-device inference (Section 2.2, Section 2.3, Section 2.4, Section 2.5, Section 2.6, Section 2.9, Section 2.11, Section 2.12 and Section 2.14). Four topics included in this review sit outside that core definition and are included as adjacent or enabling technologies rather than as continuous monitoring in the strict sense; each is flagged at first mention rather than left to blend into the core scope:

(i) Chest radiography and CT (Section 2.7) are episodic, not continuous, but are included because point-of-care imaging is frequently interpreted within an acute, time-sensitive clinical workflow. (ii) Robotic surgery (Section 2.13) is included as an example of real-time, sensor-driven monitoring applied intraoperatively rather than ambulatorily. (iii) Large language models in clinical workflows (Section 2.13) are included because LLM-based documentation and decision support increasingly sit alongside real-time monitoring dashboards in clinical settings, not because LLMs themselves perform continuous physiological sensing. (iv) 6G localisation (Section 2.10) is included as a prospective enabling technology for future real-time monitoring infrastructure, evaluated in the cited literature through simulation rather than field deployment. These four topics are treated at less depth than the wearable- and cardiac-monitoring core, and conclusions drawn from them are qualified accordingly throughout Section 4 and Section 5.

### 3.3. Source Classification and Evidence Overlap

A source was included if it (i) reported on AI, IoT, or communication technologies applied to real-time or continuous physiological or diagnostic monitoring, within the scope defined in Section 3.2; (ii) appeared as a full peer-reviewed article or systematic review with a verifiable DOI; and (iii) reported at least one quantitative outcome (accuracy, latency, power consumption, or similar) if an empirical study, or a defined search and selection methodology if it was a review. Every candidate reference was individually cross-checked against its publisher record, PubMed Central entry, or DOI resolver to confirm the author list, journal, volume, and year before inclusion; sources that could not be independently confirmed were dropped rather than kept on a partial match.

The 75 included sources are not uniformly peer-reviewed primary studies; Table 1 categorises all 75 by publication type. All 75 are peer-reviewed journal articles or conference proceedings; no indexed preprints remain in the reference list following verification. Of the 75, 19 are primary empirical studies; 32 are systematic reviews, scoping reviews, or meta-analyses with a stated search and selection methodology; 23 are narrative or general reviews without a systematic search protocol; and 1 is a methodology guideline document (the PRISMA 2020 statement, cited in Section 3.1 for terminology rather than as evidence). This mixture reflects the technology-mapping purpose of this review, which draws on primary studies for specific quantitative results and on systematic and narrative reviews for broader technological and clinical context, rather than pooling a single outcome across a homogeneous evidence base.

Because several of the systematic reviews cited here synthesise dozens of primary studies each (for example, the 80-study review by Gorce and Jacquier-Bret in Section 2.3, or the 62-study meta-analysis by Abbas et al. in Section 2.8), and because a small number of individual primary studies are also cited separately for illustrative detail (for example, Nait Aicha et al. and Mohammad et al. in Section 2.3), the same underlying participant cohorts or datasets may be represented more than once across this narrative synthesis. This review does not attempt participant-level deduplication across primary studies and the systematic reviews that may already contain them; citations here serve to map the technological and clinical landscape rather than to pool a single effect size, and Table 2 lists the primary studies discussed in enough quantitative detail to warrant individual characterisation, so that a reader can identify which specific studies contribute independent evidence.

### 3.4. Study Characteristics of the Primary Evidence

The sample sizes and validation strategies above are transcribed as reported in each source; several primary studies did not report a per-patient sample size distinct from the number of signal segments or recordings used, and this is marked accordingly rather than inferred. No formal risk-of-bias or quality-appraisal instrument (such as QUADAS-2 for diagnostic accuracy studies, or the Mixed Methods Appraisal Tool) was applied to the studies in Table 2. This is a limitation, not an oversight: the studies span retrospective model development, prospective cohorts, meta-analyses, and a regulatory document analysis, and no single appraisal instrument covers this range of designs without substantial adaptation. Applying a single instrument uniformly would have produced a false sense of comparability across designs that it was not built to assess; applying different instruments to different subsets was judged to be beyond the scope of a narrative synthesis and is noted in Section 5.4 as a direction for a future, narrower systematic review.

### 3.5. Synthesis Approach

**Figure 1:**
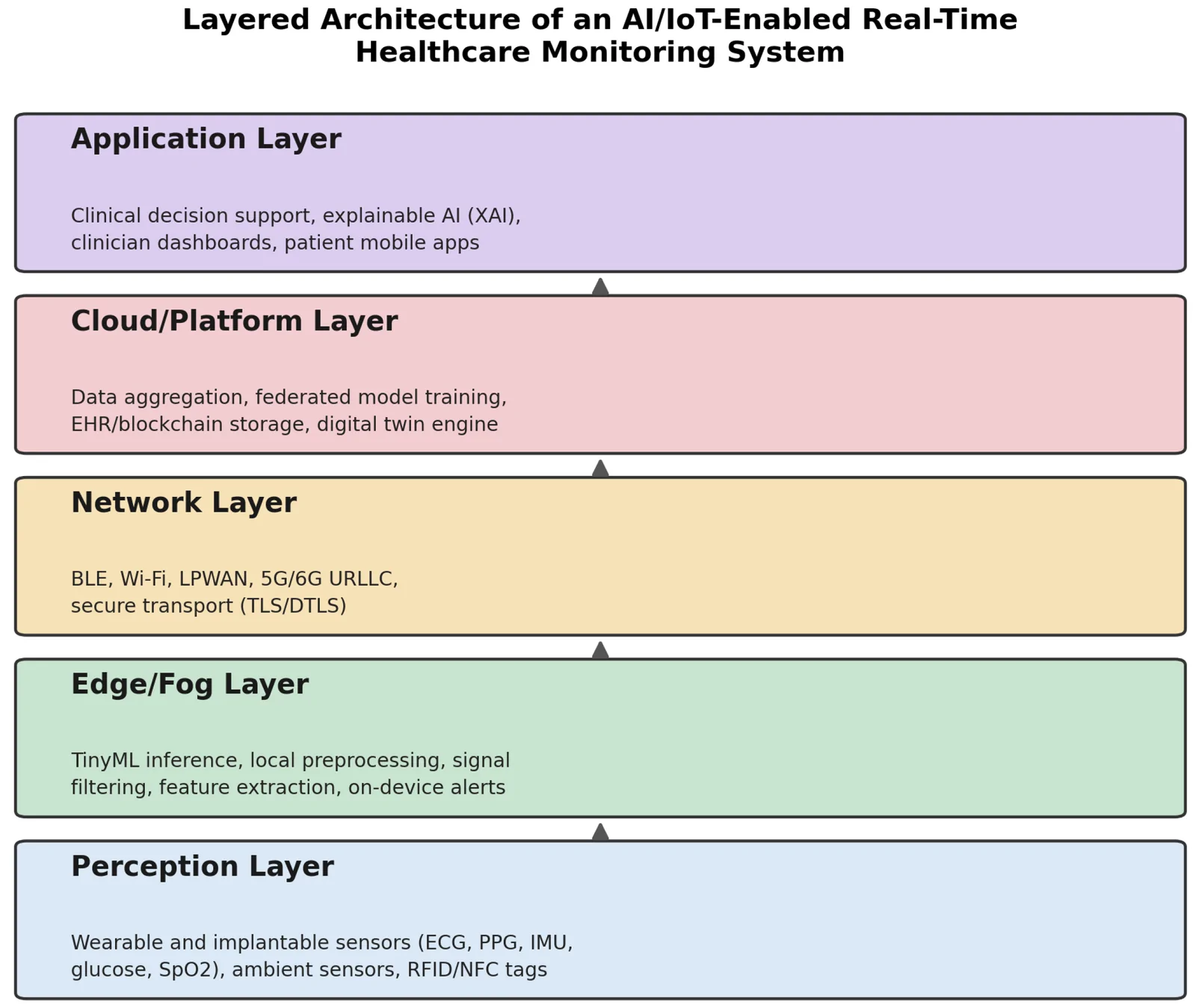
Layered architecture synthesising the perception, edge, network, cloud, and application layers described across the reviewed IoMT and wearable-sensing literature [1,2,3,10,14,16,19,38].

**Figure 2:**
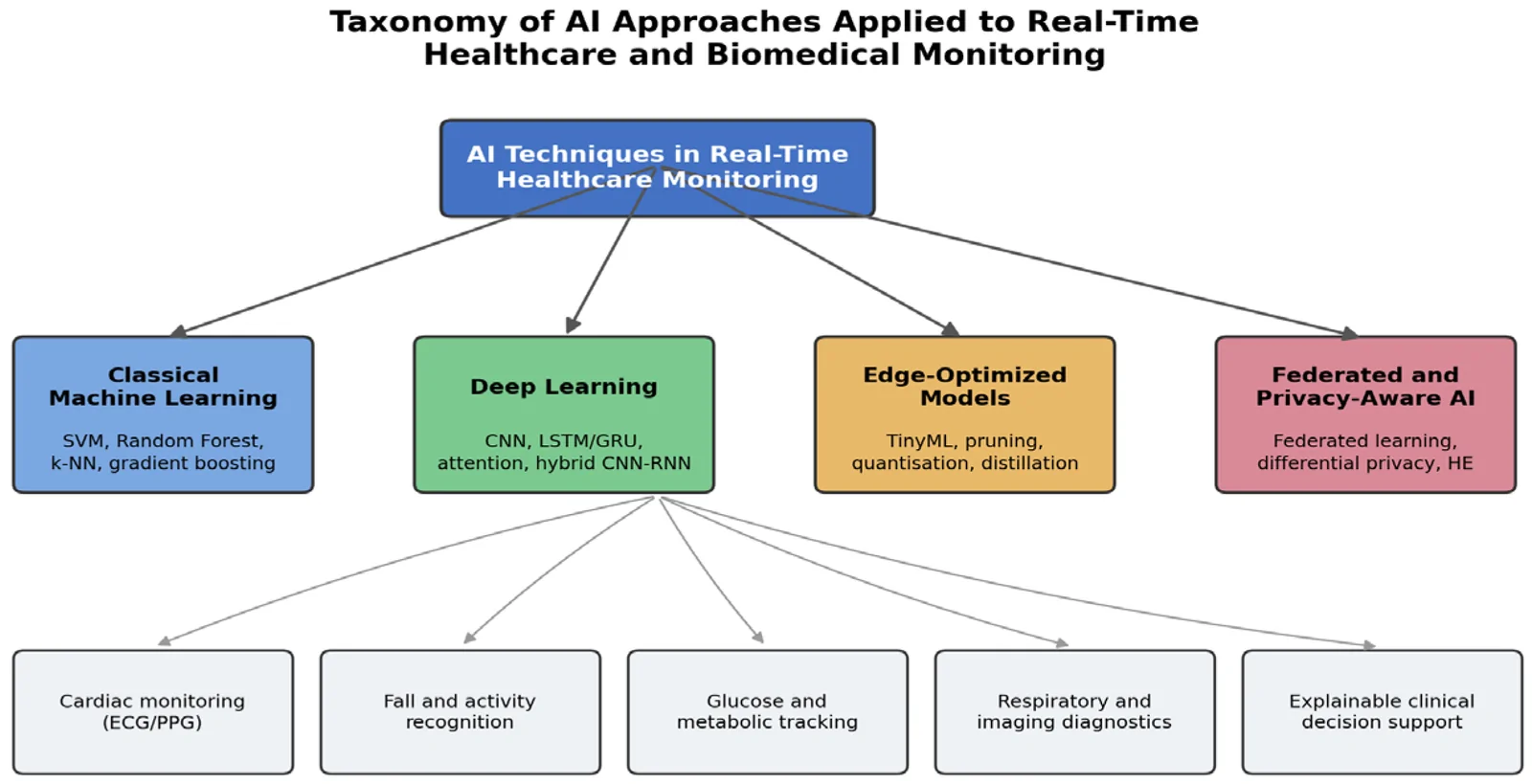
Taxonomy of AI technique families identified across the reviewed literature and their principal clinical monitoring applications.

**Figure 3:**
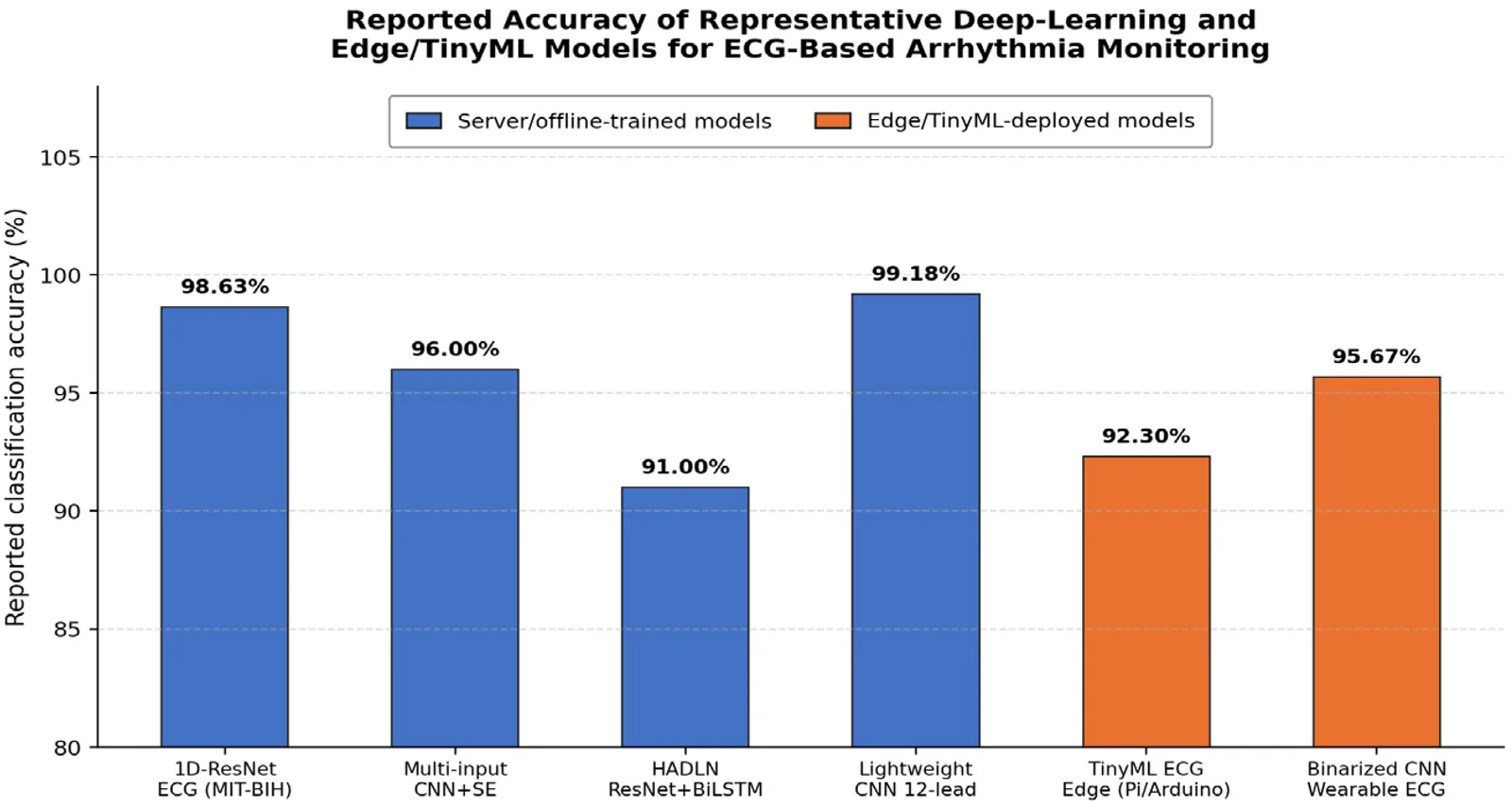
Reported accuracy of six representative ECG deep learning studies reviewed in this article (of the eight cardiac biosignal studies in Table 5), distinguishing studies with an explicit resource/hardware constraint from those without. Bai et al. [8] (multi-class AUC/F1, no single accuracy figure) and Ma et al. [11] (phonocardiogram, not ECG) are excluded from this chart because their reported metrics are not on the same accuracy scale as the other six studies; both remain in Table 5. Values are as reported in the original studies and are not the result of a controlled head-to-head benchmark [6,7,10,32,33,34].

Included sources were mapped onto a five-layer architectural taxonomy, namely, perception, edge/fog, network, cloud/platform, and application, shown in Figure 1 and discussed against existing IoMT reference architectures in Section 2.1, and onto a complementary taxonomy of AI technique families, i.e., classical machine learning, deep learning, edge-optimised models, and federated/privacy-aware AI, shown in Figure 2. Quantitative findings on cardiac biosignal classification were pulled into a structured comparison table (Table 5) and visualised together (Figure 3), so that the accuracy figures reported under different datasets and evaluation protocols could be examined side by side; this comparison is presented as an indicative summary of a heterogeneous evidence base, not as a controlled benchmark, and Section 4.4 restates this limitation explicitly. The findings on medical imaging, explainable AI, continuous glucose monitoring, connectivity/digital twins, cross-cutting applications, consumer/neurological monitoring, and materials/systems infrastructure were folded into further comparison tables (Tables 6–13) to extend the architectural analysis beyond the wearable- and cardiac-monitoring core of this review.

## 4. Results

Consistent with reviewer feedback, this section opens with the characteristics of the included evidence base (already presented in Table 1 and Table 2 in Section 3) before moving to thematic synthesis organised by architectural layer and application domain.

### 4.1. Layered Architecture of Real-Time Monitoring Systems

Pulling together the surveys discussed in Section 2.1 [1,2,3,4,5] with the device- and application-level studies in Section 2.2, Section 2.3, Section 2.4, Section 2.5, Section 2.6, Section 2.7, Section 2.8, Section 2.9 and Section 2.10, a consistent five-layer architecture emerges across the reviewed literature, shown in Figure 1. The perception layer covers wearable, implantable, and ambient sensors picking up ECG, photoplethysmography (PPG), inertial motion, glucose concentration, and diagnostic images. The edge/fog layer handles local preprocessing and, where the hardware allows, on-device inference through TinyML, as in Hizem et al.’s and Ma et al.’s edge-deployed cardiac biosignal detectors [10,11]. The network layer moves data via protocols running from Bluetooth Low Energy for short-range wearable links up to 5G URLLC and, eventually, 6G-native localisation for latency-critical remote monitoring [19,20]. The cloud/platform layer aggregates data for federated model training [14,15], secures records with blockchain [16,17,18], and may host a digital twin engine [38,39]. Finally, the application layer provides outputs to clinicians and patients, ideally through the explainable interfaces discussed in Section 2.8 [21,22]. As discussed in Section 2.1, this taxonomy is a refinement of existing IoMT reference architectures rather than a novel free-standing framework.

This layered view carries a practical lesson for system designers: privacy, latency, and interoperability each live mostly in a different layer, so a system that is weak in just one, for example, an accurate on-device model paired with an insecure cloud backend, can still fail to meet regulatory or clinical requirements overall.

### 4.2. AI Technique Taxonomy and Application Mapping

The AI methods used across the reviewed studies fall into four families, shown in Figure 2: classical machine learning, a baseline in most fall detection and glucose-monitoring studies; deep learning, the dominant approach for ECG, HAR, and imaging tasks; edge-optimised models built through pruning, quantisation, or binarisation [10,11,12,13,34]; and federated or privacy-aware AI, which modifies the training procedure rather than the architecture itself [14,15,16]. Deep learning dominates cardiac monitoring, fall/activity recognition, and imaging diagnostics. Edge-optimised variants of the same architectures are increasingly reported specifically for wearable deployment rather than as a separate line of research, and explainability mechanisms, including attention and Grad-CAM, are increasingly incorporated into the primary model rather than applied afterward [21,32] (Table 3).

### 4.3. Wearable Sensing, Fall Detection, and Human Activity Recognition

Table 4 summarises the fall detection, HAR, and general wearable-sensing studies covered in Section 2.2 and Section 2.3. Gorce and Jacquier-Bret’s systematic review, spanning 80 studies, is the largest single evidence base in this set and provides the clearest signal within the studies they screened: deep learning outperformed threshold-based and classical machine learning methods across accuracy, precision, sensitivity, specificity, and F1-score, while wearable-only configurations underperformed hybrid (wearable plus ambient) setups [28]. This finding is relevant to system design, since it indicates that wearable-only systems, despite being the most practical option for continuous free-living use, may carry a performance penalty relative to instrumented-environment alternatives that are more difficult to deploy at scale. Gu et al.’s clinical systematic review reports a comparable finding from a different perspective: predictive accuracies as high as 0.97 AUC for fall risk and mobility decline prediction, tempered by an explicit caution that small sample sizes and inconsistent methodology limit the generalisability of the 30 reviewed studies [26].

### 4.4. Cardiac Biosignal Monitoring: Accuracy Across the Server-to-Edge Spectrum

Table 5 and Figure 3 pull together the quantitative results from the eight cardiac biosignal studies covered in Section 2.4, in which seven address ECG specifically, and one addresses phonocardiogram-based heart murmur detection. The reported accuracies run from 91% for four-class rhythm classification with the attention-based HADLN model [32] up to 99.48% for a lightweight channel-attention CNN on the INCART database [33]. These figures are drawn from eight different studies using different datasets (MIT-BIH, INCART, PhysioNet 2017 Challenge, CirCor DigiScope), different class counts, different signal modalities (ECG versus PCG), and different validation protocols. This heterogeneity means that the comparison in Table 5 and Figure 3 cannot be used to conclude that server-scale models are inherently more accurate than edge-deployed ones, nor does it provide a measured “accuracy cost” of edge deployment; no controlled experiment in the reviewed literature holds the dataset, class definition, and validation protocol constant while varying only the deployment target. What can be said, more cautiously, is that within this heterogeneous set, the four studies reporting an explicit hardware or resource constraint [10,11,34] report accuracies of 85–96%, and the four studies without a stated hardware constraint report accuracies of 91–99.5%; the ranges overlap, and the difference should be read as an association observed across a small, heterogeneous set of studies rather than a controlled effect of edge deployment itself.

Hizem et al.’s edge-deployed model reports power consumption (0.024 mW at 92.3% accuracy) in addition to accuracy and is one of the few studies in this set to report an energy figure alongside accuracy [10]. Ma et al. show a comparable resource/accuracy trade-off outside ECG as well: a ten times reduction in model parameters combined with self-supervised pre-training for phonocardiogram-based valvular heart disease screening, deployed for near real-time inference on standard consumer smartphones [11], though because this is a different signal modality from the other seven studies in this subsection, it is not merged into the same accuracy comparison in Figure 3.

**Table 5:** Deep learning models for cardiac biosignal monitoring reviewed in this article. Seven studies use ECG; one (Ma et al.) uses phonocardiogram (PCG). Ordered by stated resource/hardware constraint.

Model/Study	Signal	Architecture	Dataset	Primary Metric(s)	Compute/Power	Deployment Target
Khan et al. [7]	ECG	1-D convolutional ResNet + SMOTE	MIT-BIH (5 classes)	98.63% accuracy	Not reported	Server/offline
Zheng et al. [6]	ECG	Multi-input CNN + SE attention	STFT-derived (multi-resolution)	96.0% accuracy	Not reported	Server/offline
Bai et al. [8]	ECG	CNN + BiGRU + multi-head attention	MIT-BIH, PTB	Multi-class AUC and F1-score (no single accuracy figure reported)	Not reported	Server/offline
Jiang et al. (HADLN) [32]	ECG	ResNet + Bi-LSTM + attention	PhysioNet 2017 (4 classes)	91.0% accuracy	Not reported	Server/offline
Elsheikhy et al. [33]	ECG	Lightweight CNN + channel attention	MIT-BIH/INCART	99.18%/99.48% accuracy	Reduced parameter count (lightweight)	Lightweight, 2- and 12-lead
Wang et al. [34]	ECG	Binarised CNN	MIT-BIH (5 classes)	95.67% accuracy (vs. 96.45% full-precision)	12.65× speedup; 24.8× storage reduction	Resource-constrained wearable
Hizem et al. [10]	ECG	Pruned/quantised TinyML CNN	ECG anomaly detection	92.3% accuracy	0.024 mW	Raspberry Pi/Arduino edge
Ma et al. [11]	PCG (valvular disease)	Lightweight CNN + self-supervised pre-training	Clinical PCG recordings	4–20% accuracy gain over non-SSL baseline on noisy data	10× fewer parameters; ~1/3 power of full CNN	Smartphone (0.03–0.37 s inference)

### 4.5. Edge Computing, TinyML, and Privacy-Preserving Collaborative Learning

Table 6 summarises this review’s coverage of edge/TinyML deployment and the privacy-preserving learning mechanisms from Section 2.5 and Section 2.6. A consistent pattern across the FL and blockchain literature is that no single mechanism satisfies every regulatory requirement independently. FL prevents raw data from being centralised but remains vulnerable to model-inversion and poisoning attacks unless combined with a cryptographic or blockchain-based safeguard [14,16]; blockchain secures record integrity and access control but does not by itself reduce the computational cost of training [17,18]. This complementarity underlies the hybrid cloud/platform layer proposed in Figure 1, in which federated training and blockchain-secured storage function as distinct mechanisms rather than substitutes for one another; as stated in Section 2.6, neither mechanism constitutes regulatory compliance independently. Heydari and Mahmoud’s broader TinyML survey provides additional context for the ECG-specific figures in Table 5, comparing the 0.18–300 ms inference latencies typical of TinyML deployments against the 10–500 ms threshold commonly cited for real-time responsiveness [13].

### 4.6. Medical Imaging Diagnostics

Table 7 summarises the medical imaging studies from Section 2.7. Both studies concentrate on chest imaging, consistent with the field’s overall post-COVID-19 emphasis, and both indicate that hybrid architectures, for example, a customised or ensembled feature extractor paired with pre-trained backbones, achieve more consistent accuracy than purely end-to-end single-network designs in the studies reviewed [9,35]. This is consistent with the hybrid architecture pattern observed in the ECG literature in Section 4.4, a similarity that may reflect a general property of deep learning applied to biomedical signals and images, although this has not been tested directly across modalities. As noted in Section 3.2, chest imaging is included here as an episodic but time-critical point-of-care application rather than as continuous monitoring in the strict sense.

### 4.7. Explainable AI and Continuous Glucose Monitoring

Table 8 summarises the explainable AI and continuous glucose monitoring literature from Section 2.8 and Section 2.9. These two subfields address different clinical problems but share a common translational barrier: a gap between offline predictive performance and the longitudinal, real-world validation routine clinical use would require. Abbas et al.’s meta-analysis of 62 XAI-in-CDSS studies and Kapoor and Hasija’s meta-analysis of ten CGM prediction studies both report declining reliability as the task moves further from the controlled conditions under which the underlying model was trained, whether through an extended glucose prediction horizon [36] or a clinical domain in which interpretability tooling remains immature [21].

### 4.8. Connectivity and Digital Twins

Table 9 summarises the network layer and digital twin literature from Section 2.10. Batool’s 5G-URLLC-integrated deep learning architecture is distinguished by a sustained multi-month evaluation across 1000 patients rather than a short benchmark, which strengthens the confidence in the reported stability under the conditions tested [19]. As stated in Section 2.10, Trevlakis et al.’s treatment of 6G-native localisation remains a simulation-based, pre-deployment capability rather than a field-validated one [20]. Khoshfekr Rudsari et al. and Banoub et al., in their respective reviews of digital twins, report some of the more notable individual clinical outcomes identified across this set of sources: a documented reduction of over 13% in cardiac arrhythmia recurrence after digital-twin-guided treatment planning in one study [38], and improved glaucoma and age-related macular degeneration risk assessment through DT-integrated ophthalmic data [39]. Both technologies remain predominantly at the modelling or early evaluation stage, and computational scalability and prospective validation remain open barriers to wider clinical translation.

### 4.9. Cross-Cutting Applications and Security Threat Model

**Figure 4:**
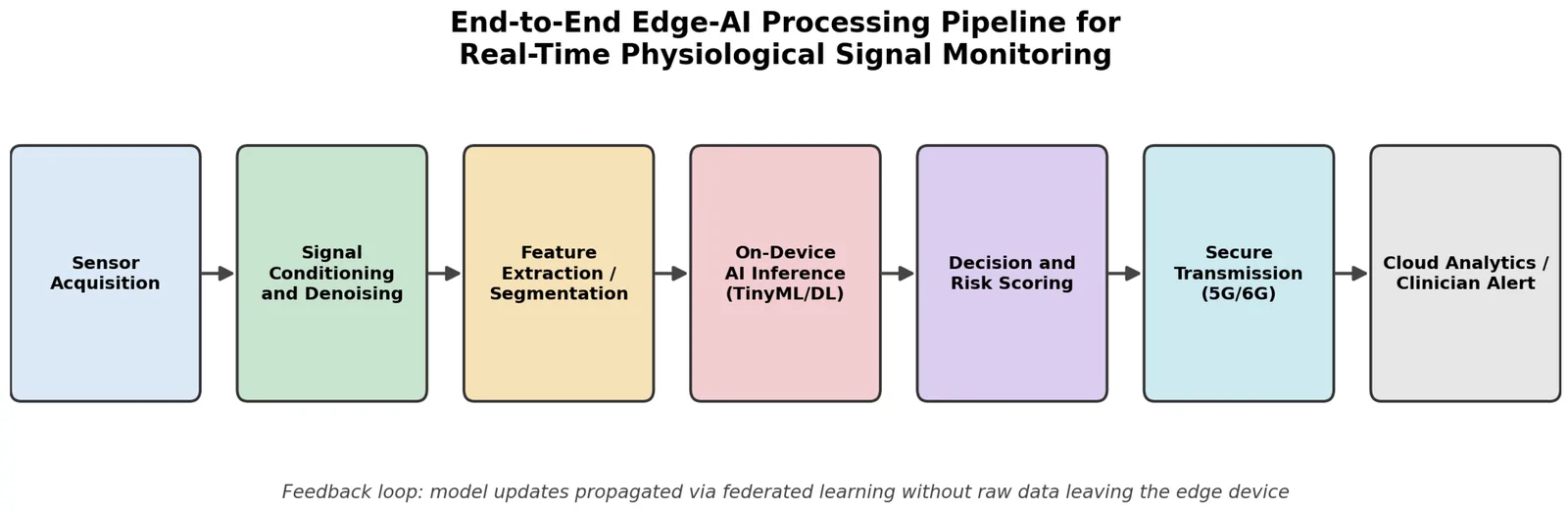
End-to-end edge-AI processing pipeline synthesised from the on-device and TinyML deployment studies reviewed in Section 4.4 and Section 4.5 [10,11,12,14].

Table 10 presents studies that do not fit neatly into the sensing-modality-organised subsections above but that bear directly on the real-time monitoring architecture developed throughout this review, either structurally, through shared technical challenges, or operationally, through the regulatory and workflow context in which the systems discussed here would need to be deployed. Ayaz et al.’s systematic literature review of the HL7 Fast Healthcare Interoperability Resources (FHIR) standard [76], covering 80 included articles, addresses a problem that affects every layer of the architecture in Figure 1: without a shared data exchange standard, the wearable, IoMT, and cloud/platform layers discussed separately throughout this review cannot be connected into a single working system [77]. Almarie et al.’s analysis of FDA-authorised ML-enabled devices and Li et al.’s review of hospital cyber-attacks indicate, from different perspectives, that the technical capability demonstrated throughout Section 4.1, Section 4.2, Section 4.3, Section 4.4, Section 4.5, Section 4.6, Section 4.7 and Section 4.8 outpaces the regulatory transparency and security posture required for safe deployment at scale [57,58]. Gao et al.’s review of medical image segmentation and Knudsen et al.’s review of AI in robotic surgery, both included as adjacent technologies per Section 3.2, extend the imaging and edge-AI themes of this review into sub-tasks—region delineation and intraoperative guidance—that fall outside the main scope of Section 4.4, Section 4.5 and Section 4.6 but draw on the same underlying architectures [59,60] (Figure 4).

Table 11 addresses the cybersecurity threat model outlined in Section 2.1 directly, mapping distinct categories of attack onto the five architectural layers rather than treating security as a single undifferentiated risk.

### 4.10. Consumer-Grade and Neurological Monitoring

Table 12 summarises the consumer cardiac, contactless-sensing, and neurological/psychiatric monitoring studies reviewed in Section 2.11 and Section 2.12. Considered together, these studies show the same server-to-edge and detection-to-prediction gradients already established for ECG and fall detection extending into new modalities: consumer smartwatch ECG performs strongly for atrial fibrillation specifically in the pooled meta-analytic evidence [40], cuffless blood pressure approaches but has not consistently been shown to reach that level [42], and neurological applications ranging from sleep apnoea to stroke consistently report that detecting an event is a more mature problem than predicting one in advance [44,45,46,47].

### 4.11. Materials, Systems, and Acute Care Applications

Table 13 covers the sensing materials, healthcare systems, and acute care studies reviewed in Section 2.13. These studies address enabling infrastructure rather than a single clinical task: energy-autonomous sensing [49,50], non-invasive sensing alternatives to implanted CGM [51], the home and hospital environments in which monitoring is deployed [52,53], specialised clinical populations [54,55,63,64], and the evidentiary and respiratory-sensing gaps that recur across this literature [56,62,65]. Taken together with Table 3, Table 4, Table 5, Table 6, Table 7, Table 8, Table 9, Table 10, Table 11 and Table 12, this final set of studies is consistent with, though it does not on its own prove, an interpretation that recurs across this review: within the evidence considered here, the sensing and AI components of real-time healthcare monitoring generally appear more technically mature than the surrounding validation, regulatory, and deployment infrastructure needed to put them into routine practice.

## 5. Discussion

### 5.1. What the Evidence Supports

A handful of observations hold up reasonably well across the individual studies reviewed here, though, as stated throughout Section 4, these are patterns observed within a heterogeneous, non-exhaustively sampled literature rather than pooled, statistically established effects. Deep learning models report high accuracy on cardiac biosignal classification tasks, generally above 90% and frequently above 95% within the eight studies reviewed in Section 4.4 [6,7,8,10,11,32,33,34]; a share of that reported accuracy is preserved under the compression used for on-device, battery-powered operation in the studies reviewed here [10,11,34], though Table 5 shows this is drawn from different datasets and validation protocols rather than a controlled comparison. Deep-learning-based fall detection and chest radiograph classification are each reported to outperform threshold-based or purely end-to-end alternatives within the individual studies and reviews covered here [28], though the largest systematic review in this set indicates that wearable-only sensing trails hybrid sensing configurations specifically on the fall detection task within the 80 studies it screened. No single privacy or security mechanism, for example, federated learning, blockchain, or on-device inference, is presented as sufficient on its own in the literature reviewed; these are consistently treated as complementary technical mechanisms rather than substitutes for one another, and, as stressed in Section 2.6 and Section 4.5, none of them constitutes regulatory compliance by itself [14,15,16,17,18]. Furthermore, model interpretability and continuous-monitoring reliability appear, across several unrelated subfields, to degrade under broadly similar conditions, longer prediction horizons, less-studied clinical domains, and less-controlled deployment settings, whether the underlying task is glucose forecasting or clinical decision explanation [21,36].

### 5.2. Detection, Classification, Prediction, Diagnosis, and Risk Assessment: A Necessary Distinction

This review’s evidence base spans several clinically distinct tasks that are easy to conflate under a general label such as “monitoring performance”. Detection asks whether an event is happening now (a fall, a seizure, an arrhythmia in progress). Classification asks which category an already-detected event belongs to (which arrhythmia type, which lung finding). Prediction asks whether an event will happen in the future, before any direct signal of it is present (a freezing-of-gait episode, a stroke, a sepsis onset). Diagnosis asks whether a patient has an underlying condition, generally integrating multiple signals and clinical context beyond a single sensor stream. Risk assessment asks about a longer-horizon probability or subphenotype rather than a discrete event. These are not interchangeable, and the evidence quality differs sharply across them in this review’s own findings: detection and classification tasks (Section 4.4 and Section 4.6) are comparatively well studied and report high accuracy; prediction tasks (Section 2.12: freezing-of-gait, stroke) are consistently reported as the harder, less mature problem within the same reviews that report strong detection performance [46,47]; and risk-assessment or subphenotyping tasks (Section 2.9’s metabolic subphenotyping) occupy a middle ground, with promising but not yet clinically deployed accuracy [37]. Claims in this review about “high accuracy” should be read against this distinction rather than as a single undifferentiated capability level.

### 5.3. Open Challenges

Several limitations recur consistently across the reviewed studies. Reported accuracies for ECG, fall detection, and imaging models are rarely directly comparable across studies, since datasets, class definitions, and evaluation protocols differ; the comparison in Table 5 and Figure 3 should be read as indicative of the achievable range, not a ranking on a common benchmark, and not as evidence that server-scale models are inherently superior to edge-deployed ones. Prospective, real-world clinical validation remains comparatively rare relative to retrospective evaluation on public datasets such as MIT-BIH. Batool’s multi-month, multi-patient evaluation is the exception rather than the norm [19], and Abedi et al.’s finding that only 19 of 153 candidate cardiovascular-monitoring studies met scoping review inclusion criteria for genuinely real-time, validated deployment indicates the distance remaining before routine clinical translation [25]. Federated learning research in healthcare remains concentrated on privacy mechanisms relative to fairness and governance, a gap the systematic review of FL ethics identifies explicitly [15]. Explainability research and glucose-monitoring research show a similar horizon-dependent decline in reliability: longer prediction windows and less-mature clinical domains both correspond to weaker, less validated evidence [21,36], suggesting that the validation horizon length may be a useful basis for comparing studies across otherwise unrelated areas of this literature.

### 5.4. Limitations of the Current Evidence Base

Beyond the open challenges discussed above, several structural limitations run through the underlying literature and through this review’s own methodology and are stated together here. First, this is a narrative review without a PRISMA-guided search protocol or a flow diagram of records identified, screened, and excluded; Section 3.1 explains why a flow diagram was not fabricated for a search process that was iterative rather than database-driven, and this means the coverage in Section 2 and Section 4 should not be read as exhaustive. Second, no formal risk-of-bias or quality appraisal instrument was applied to the primary studies in Table 2, for the reasons given in Section 3.4; readers should not assume that the primary studies in this review have been screened for methodological quality beyond the eligibility criteria in Section 3.3. Third, most of the systematic reviews cited throughout this article, however methodologically rigorous individually, cover only a narrow evidence base: sample sizes in the primary studies they summarise are frequently small, and several of the reviews explicitly flag this as their principal limitation. Fourth, prospective, real-world validation remains the exception rather than the rule; Batool’s multi-month, multi-patient evaluation [19] and Frodi et al.’s six-month wearable-adherence study [69] stand out precisely because sustained, real-world deployment data of this kind is uncommon elsewhere in the literature. Fifth, the field lacks standardised, shared benchmarks for several of its core tasks, which is why the comparisons in Table 5 and Figure 3 are presented as indicative rather than as a controlled benchmark. Sixth, data quality and completeness are rarely reported in detail; Chakrabarti et al.’s work on missing data imputation [70] and Frodi et al.’s adherence findings [69] both suggest that accuracy figures from controlled study conditions may not transfer cleanly to real-world, intermittently missing data. Seventh, this review is subject to publication bias in the underlying literature. Positive or high-accuracy results are more likely to be published than null or negative findings, and this review has not attempted to quantify or correct for that bias, since doing so would require access to unpublished or null-result studies outside the scope of the search described in Section 3.1. Eighth, most of the reviewed evidence is published in English and in venues with a persistent DOI, which likely under-represents relevant non-English-language research. Finally, several sub-fields discussed here, specifically edge generative AI [74] and LLM-based clinical workflows [61] among them, are moving quickly enough that specific figures reported in the cited studies may already be dated by the time this review is read.

### 5.5. Future Directions

The following are proposed directions for future research, presented separately from the evidence-supported observations in Section 5.1 and Section 5.2 because they extend beyond what any single study or the aggregate evidence base directly demonstrates. Standardised, shared benchmarks for ECG, fall detection, and chest imaging tasks, analogous to the informal role that MIT-BIH already occupies for arrhythmia classification, would render the comparison attempted in Table 5 considerably more rigorous. Combined FL-plus-blockchain architectures, already proposed at a conceptual level [16], would benefit from evaluation on realistic multi-institution biomedical data rather than the more general distributed-learning benchmarks that dominate the underlying FL literature. Interpretability research could usefully investigate explanation embedded within the model architecture itself; the attention mechanisms already used in several of the ECG models covered here provide one example [6,32,33], as a complement to post hoc explanation methods that the XAI literature itself identifies as difficult for clinicians to use in practice [21,22]. A subsequent, more narrowly scoped systematic review with a registered protocol, database-level search, and formal risk-of-bias appraisal focused on a single task such as ECG arrhythmia classification would be positioned to support stronger, pooled quantitative claims than this narrative synthesis can offer. The digital twin and 6G localisation literature reviewed here [20,38,39] suggest a possible future extension of the layered architecture in Figure 1 to incorporate a patient-specific modelling and native localisation layer, contingent on the computational, data integration, and network deployment barriers noted in Section 2.10 and Section 4.8; this is presented as a plausible direction rather than a predicted outcome.

## 6. Conclusions

Within the evidence reviewed here, real-time healthcare monitoring for the specific tasks studied does not appear to be limited primarily by sensing or model accuracy. The more persistent problems observed across the technologies surveyed instead concern the interfaces between architectural layers: whether a compressed model retains sufficient accuracy on an edge device, whether a prediction is presented in a form clinicians are willing to act on, and whether patient data can move safely between institutions whose systems were not designed to interoperate. Cardiac biosignal classification illustrates this pattern: accuracy in the low-90s to high-90s range is reported both on powerful servers and, increasingly, on the wearable itself across the eight studies reviewed in Section 4.4, though these figures come from different datasets and are not a controlled comparison.

The most consistent pattern identified in this review is complementarity rather than substitution. Federated learning and blockchain address different aspects of the same governance problem—one limiting data centralisation, the other securing the resulting records—and neither is a substitute for the other or a guarantee of regulatory compliance. Wearable-only sensing and hybrid ambient-plus-wearable sensing trade off practicality against reported accuracy in the fall detection literature specifically, without either configuration being unambiguously superior across the full evidence base. As Section 5.2 sets out, detection, classification, prediction, diagnosis, and risk assessment are distinct clinical tasks with different levels of evidentiary maturity in this review’s own findings; claims regarding accuracy should be interpreted with this distinction in mind.

The evidence reviewed here suggests that the principal barrier to routine clinical use is as much a gap in evidence and infrastructure as a gap in capability: prospective, real-world validation remains the exception across nearly every application area covered, from cardiac wearables to oncology monitoring to paediatric biosensors, and studies reporting sustained, multi-month, real-patient deployment are correspondingly uncommon. A monitoring architecture that couples lightweight, privacy-preserving on-device inference with interoperable, auditable cloud infrastructure, and that treats prospective validation as a primary requirement rather than a subsequent step, is consistent with the evidence reviewed here; it is proposed as a direction for future work rather than presented as an established finding of this review. Addressing the evidentiary gap identified in Section 5—through registered systematic reviews of individual tasks, prospective multicentre validation, and standardised, appraised benchmarking—is likely to contribute more to clinical translation than further retrospective accuracy improvements on existing public datasets.

## Figures and Tables

**Table 2:** Characteristics of primary empirical studies discussed in quantitative detail in this review.

Study	Design	Dataset/Population	Sample Size	Validation	Deployment Setting
Khan et al. [7]	Retrospective model development	PhysioNet MIT-BIH ECG recordings	48 recordings (5 heartbeat classes)	10-fold cross-validation	Server/offline
Zheng et al. [6]	Retrospective model development	STFT-derived ECG (multi-resolution)	Not stated per-patient in source	Held-out test set	Server/offline
Jiang et al. [32]	Retrospective model development	PhysioNet 2017 Challenge (4 classes)	8528 ECG recordings	Held-out test set	Server/offline
Bai et al. [8]	Retrospective model development	MIT-BIH + PTB Diagnostic ECG	Combined multi-database	Held-out test set	Server/offline
Elsheikhy et al. [33]	Retrospective model development	MIT-BIH and INCART	Combined multi-database	Held-out test set	Lightweight, 2- and 12-lead
Wang et al. [34]	Retrospective model development	MIT-BIH (5 classes)	Not stated per-patient in source	Held-out test set	Resource-constrained wearable
Hizem et al. [10]	Retrospective model development + on-device test	ECG anomaly detection dataset	Not stated per-patient in source	Held-out test set	Raspberry Pi/Arduino edge
Ma et al. [11]	Retrospective model development + on-device test	Clinical phonocardiogram recordings (PCG)	Labelled + unlabelled PCG corpus	Held-out test set; SSL pre-training	Smartphone (consumer-grade)
Nait Aicha et al. [29]	Prospective cohort	Trunk accelerometer, older adults	*n* = 296	Train/test split	Free-living, daily life
Mohammad et al. [30]	Retrospective model development	Waist accelerometer + gyroscope	Not stated per-patient in source	Held-out test set	Wearable, pre-impact
Shahid et al. [40]	Diagnostic meta-analysis	Apple Watch ECG vs. 12-lead ECG	11 studies, 4241 participants	Pooled sensitivity/specificity	Consumer wearable
Liu et al. [42]	Retrospective model development	MIMIC-IV (PPG)	Public critical care database	Held-out test set	Cuffless, PPG-based
Frodi et al. [69]	Prospective cohort	SafeHeart ICD population	Cohort study, 6-month follow-up	Longitudinal adherence tracking	Implantable device + wearable
Metwally et al. [37]	Prospective cohort	At-home OGTT + CGM	Cohort study	Cross-validation, subphenotype classification	At-home CGM
Nguyen et al. [63]	Before-and-after study	Multiple critical care units	Multi-unit before/after comparison	Pre/post outcome comparison	Virtual ICU/tele-critical-care
Sang et al. [65]	Retrospective model development	Accelerometer patch, respiratory sounds	Not stated per-patient in source	Held-out test set	Wearable patch
Al-Sheikh et al. [9]	Retrospective model development	Chest X-ray and CT (COVID-19/pneumonia)	Not stated per-patient in source	Held-out test set	Server/offline
Almarie et al. [57]	Cross-sectional regulatory analysis	FDA 2024 device authorisations	All 2024 ML-enabled device summaries	Not applicable (document analysis)	Regulatory/not a clinical deployment

**Table 3:** IoMT architectural layers and their treatment in the reviewed survey literature.

Layer	Primary Function	Representative Technologies	Reviewed In
Perception	Physiological/imaging signal acquisition	ECG/PPG electrodes, IMU, glucose biosensors, radiography	[1,9,23,24]
Edge/Fog	Local preprocessing and inference	TinyML, pruning, quantisation, binarisation	[10,11,12,13]
Network	Data transport and localisation	BLE, Wi-Fi, 5G URLLC, 6G positioning	[2,19,20]
Cloud/Platform	Aggregation, training, storage	Federated learning, blockchain ledgers, digital twins	[14,15,16,17,18,38]
Application	Clinical/patient interface	Dashboards, XAI overlays, digital twin visualisation	[1,21,22]

**Table 4:** Wearable-sensing, fall detection, and human activity recognition studies included in this review.

Study	Sensor/Data Source	Method	Key Reported Finding
Gorce & Jacquier-Bret [28]	Wearable, ambient, hybrid (80 studies)	PRISMA systematic review	Deep learning outperformed threshold/ML; hybrid sensing outperformed wearable-only
Nait Aicha et al. [29]	Trunk accelerometer (*n* = 296)	CNN, LSTM, ConvLSTM vs. biomechanical baseline	Learned features matched/exceeded hand-engineered biomechanical features
Mohammad et al. [30]	Waist-worn accelerometer + gyroscope	CNN-RNN ensemble	Pre-impact detection framework for airbag-based injury prevention
Gu et al. (HAR survey) [31]	Multimodal wearable/mobile sensors	Deep learning survey	Labelled data cost and on-device deployment are the main open problems
Abedi et al. [25]	Wearable cardiovascular sensors	AI scoping review (19/153 studies)	Real-time cardiovascular monitoring research remains limited and under-validated
Gu et al. (clinical review) [26]	IMU, smartwatch, multi-sensor	PRISMA systematic review (30 studies)	AUC up to 0.97 for fall/mobility risk; small samples limit generalisability
Aruleba [27]	ECG, PPG, IMU, EDA wearables	Narrative deployment pathway review	Proposes validation ladder and reliability toolkit for edge model updates

**Table 6:** Edge/TinyML deployment and privacy-preserving learning mechanisms reviewed in this article.

Mechanism	Representative Study	Primary Benefit	Primary Limitation Reported
TinyML on-device inference	Elhanashi et al. [12]; Heydari & Mahmoud [13]	Eliminates continuous raw data transmission; sub-300 ms latency	Reduced model capacity vs. server-scale models
Federated learning (general)	Abbas et al. [14]	Trains shared models without centralising patient data	Vulnerable to poisoning/model-inversion attacks; not a compliance guarantee
Federated learning (ethics-focused)	Mir et al. [15]	Structures fairness and governance considerations	Fairness/governance less studied than privacy mechanisms
FL + blockchain	Qammar et al. [16]	Blockchain secures FL aggregation and provides audit trail	Added transaction/communication overhead
Blockchain-based EHR	Ettaloui et al. [17]; Jayapriya & Jeyanthi [18]	Decentralised storage; fine-grained access control	Throughput and storage overhead at scale; not a compliance guarantee

**Table 7:** Deep learning studies for medical imaging diagnostics reviewed in this article.

Study	Modality/Task	Method	Key Reported Finding
Al-Sheikh et al. [9]	Chest X-ray and CT; COVID-19/pneumonia/normal	Custom CNN + AlexNet + VGG16 ensemble, novel image enhancement	Pre-trained ensemble with tailored enhancement improved multi-class discrimination
Meedeniya et al. [35]	Chest X-ray; pneumonia/COVID-19	Systematic review of DL classification approaches	Hybrid CNN-feature + robust classifier outperformed pure end-to-end networks

**Table 8:** Explainable AI and continuous glucose monitoring studies reviewed in this article.

Study	Domain	Method	Key Reported Finding
Abbas et al. [21]	XAI in clinical decision support (62 studies)	Systematic meta-analysis	Grad-CAM and attention dominate; longitudinal validation remains scarce
Xu et al. [22]	CDSS interpretability, 2011–2020	Systematic review	Ante hoc vs. post hoc choice depends on data type and clinician/patient needs
Kapoor & Hasija [36]	CGM + IoT glucose prediction (10 studies)	Meta-analysis across 15–60 min horizons	RMSE rises from 15.02 to 35.89 mg/dL as horizon lengthens; Random Forest strongest
Metwally et al. [37]	At-home OGTT + CGM metabolic phenotyping	ML subphenotype classification	AUC 88–95% for muscle/hepatic/beta-cell/incretin subphenotypes

**Table 9:** Connectivity and digital twin studies reviewed in this article.

Technology	Study	Reported Outcome	Noted Limitation
5G URLLC-integrated deep learning	Batool [19]	Stable prediction accuracy and reduced latency over a 3-month, 1000-patient evaluation	Depends on 5G URLLC coverage availability
6G-native localisation	Trevlakis et al. [20]	High-accuracy indoor/on-body positioning as a native network capability	Technology still pre-deployment; simulation-based evidence only
Digital twin (cardiology/personalised medicine)	Khoshfekr Rudsari et al. [38]	>13% reduction in cardiac arrhythmia recurrence in one digital-twin-guided case series	Computational scalability; limited prospective validation
Digital twin (ophthalmology)	Banoub et al. [39]	Improved glaucoma/AMD risk assessment and surgical planning	Data integration and privacy concerns limit clinical translation

**Table 10:** Cross-cutting and emerging application studies reviewed in this article.

Study	Application Domain	Relevance to Real-Time Monitoring Architecture
Ayaz et al. [77]	HL7 FHIR interoperability standard	Shared data exchange standard connecting perception, edge, and cloud layers
Almarie et al. [57]	FDA regulation of ML-enabled devices	Regulatory transparency gap alongside clinical validation gaps
Li et al. [58]	Hospital cybersecurity	Security posture needed for safe IoMT/blockchain deployment
Gao et al. [60]	Medical image segmentation	Region-delineation extension of imaging diagnostics (adjacent technology)
Knudsen et al. [59]	AI in robotic surgery	Real-time, sensor-driven monitoring applied intraoperatively (adjacent technology)

**Table 11:** Cybersecurity threat categories mapped onto the five-layer architecture.

Threat Category	Primary Layer(s)	Description	Relevant Discussion
Data interception/eavesdropping	Network	Unencrypted or weakly encrypted transmission intercepted in transit	[4]
Device compromise/physical tampering	Perception	Direct compromise of a wearable or implantable sensor’s firmware or hardware	[4]
Data/model poisoning	Cloud/Platform	Malicious training data or updates injected during federated training	[14]
Model-inversion/membership-inference	Cloud/Platform	Attempts to reconstruct or infer training data from model parameters or updates	[14]
Adversarial perturbation	Edge/Application boundary	Crafted input designed to trigger an incorrect inference at deployment time	[13]
Ledger/consensus-layer attacks	Cloud/Platform	Attacks targeting blockchain consensus or smart-contract logic in EHR systems	[17,18]

**Table 12:** Consumer-grade cardiac sensing, contactless capture, and neurological/psychiatric monitoring studies reviewed in this article.

Study	Domain	Method/Scope	Key Reported Finding
Shahid et al. [40]	Consumer ECG (AFib)	Meta-analysis, 11 studies, 4241 participants	94.8% sensitivity, 95% specificity vs. 12-lead ECG
Liu et al. [42]	Cuffless blood pressure	ResNet-BiLSTM on MIMIC-IV	MAE 3.47/2.81 mmHg; meets AAMI/BHS Grade A
Kebe et al. [43]	Contactless vital signs	CW/UWB radar review	Matches contact-sensor accuracy for children/elderly
Abd-Alrazaq et al. [44]	Sleep apnoea	Meta-analysis, 38 studies	Accuracy highly sensor-placement- and metric-dependent
Singh et al. [45]	Epileptic seizure detection	Narrative review: EEG/MRI/wearables	Real-time detection now feasible on implants/wearables
Pardoel et al. [46]	Parkinson’s freezing of gait	Review, 74 studies	Detection mature; prediction remains a harder problem
Alhakeem et al. [47]	Stroke risk prediction	Systematic review, 5 studies	Small evidence base for real-time pre-event prediction
Abd-alrazaq et al. [48]	Anxiety and depression	Scoping review, 69/1203 studies	Diagnosis dominates; treatment applications rare

**Table 13:** Materials, healthcare systems, and acute care studies reviewed in this article.

Study	Domain	Key Reported Finding
Shao et al. [49]	Textile physiological sensors	Rising publication trend; garment-integrated sensing maturing
Rayegani et al. [50]	Self-powered wearable sensors	Hybrid piezoelectric/triboelectric nanogenerators reduce charging burden
Martins et al. [51]	Non-invasive glucose sensing	Optical/microwave methods still trail subcutaneous CGM accuracy
Zieni et al. [52]	Ambient assisted living	Privacy/cost/usability/reliability form a persistent trade-off
Tan et al. [53]	RPM care-transition outcomes	29 studies, 16 countries: lower readmission, LOS, and cost
Cloß et al. [54]	Oncology wearables	112 studies; only 9.8% RCTs, mostly early feasibility stage
Sarpong et al. [55]	Smart wound dressings	Earlier infection detection than visual inspection; validation lags
Motahari-Nezhad et al. [56]	Digital biomarkers	RCT evidence concentrated in activity and cardiac rhythm biomarkers
Moor et al. [62]	Sepsis prediction (ICU)	Multi-marker models outperform single-marker; onset definition heterogeneity
Nguyen et al. [63]	Virtual ICU implementation	Nurse-led continuous monitoring improved clinical outcomes
Senechal et al. [64]	Neonatal vital sign monitoring	60 studies; mostly single vital sign, offline processing
Sang et al. [65]	Respiratory wheeze detection	95% accuracy, AUC 0.99, accelerometer patch outperforms baseline

## Data Availability

No new data were created or analyzed in this study. Data sharing is not applicable to this article.

## Abbreviations

The following abbreviations are used in this manuscript:

AI	Artificial Intelligence
IoMT	Internet of Medical Things
ECG	Electrocardiogram
PPG	Photoplethysmography
PCG	Phonocardiogram
CNN	Convolutional Neural Network
LSTM/BiLSTM	(Bidirectional) Long Short-Term Memory network
GRU/BiGRU	(Bidirectional) Gated Recurrent Unit
FL	Federated Learning
TinyML	Tiny Machine Learning
XAI	Explainable Artificial Intelligence
CGM	Continuous Glucose Monitoring
OGTT	Oral Glucose-Tolerance Test
URLLC	Ultra-Reliable Low-Latency Communication
HIPAA	Health Insurance Portability and Accountability Act
GDPR	General Data Protection Regulation
EHR	Electronic Health Record
PRISMA	Preferred Reporting Items for Systematic Reviews and Meta-Analyses
FHIR	Fast Healthcare Interoperability Resources
AAMI	Association for the Advancement of Medical Instrumentation
QUADAS-2	Quality Assessment of Diagnostic Accuracy Studies, version 2
